# Exploring curcumin and rosmarinic acid as potential antidotes for pesticide-induced harm to honey bees

**DOI:** 10.3389/finsc.2025.1673140

**Published:** 2025-10-22

**Authors:** Saeed Mohamadzade Namin, Tekalign Begna, Youngrak Kang, Daniel Bisrat, Arezoo Najarpoor, Delgermaa Ulziibayar, Mohammad Vatanparast, Chuleui Jung

**Affiliations:** ^1^ Agriculture Research Institute, GyeongKuk National University, Andong, Republic of Korea; ^2^ Department of Horticulture, College of Agricultural Science, Oregon State University, Corvallis, OR, United States; ^3^ Department of Plant Medicals, GyeongKuk National University, Andong, Republic of Korea; ^4^ Department of Pharmaceutical Chemistry and Pharmacognosy, School of Pharmacy, College of Health Sciences, Addis Ababa University, Addis Ababa, Ethiopia; ^5^ Institute for Biosafety in Plant Biotechnology, Julius Kühn Institute (JKI), Federal Research Centre for Cultivated Plants, Quedlinburg, Germany

**Keywords:** *Apis mellifera*, insecticide, pollinator health, phenolic compound, detoxification, toxicity

## Abstract

Honey bees are essential pollinators in global food production, however, their populations are increasingly threatened by insecticides. Protecting bees from these chemical stressors is critical not only for ecosystem stability but also for agricultural sustainability. Natural dietary compounds, such as curcumin (CU) and rosmarinic acid (RA), have demonstrated antioxidant and detoxification-promoting properties in other organisms and may offer a promising approach to enhancing honey bee resilience to pesticide exposure. This study investigates the potential of CU and RA to mitigate pesticide-induced harm in honey bees. In acute toxicity tests, newly emerged bees and foragers were topically exposed to lethal doses of acetamiprid (1.04 µg/bee for newly emerged and 15.3 µg/bee for forager), carbaryl (0.06 µg/bee for newly emerged and 0.51 µg/bee for forager), and flupyradifurone (15.6 µg/bee for newly emerged and 24.1 µg/bee for forager), followed by post-feeding with CU and RA at 50, 100, and 200 ppm for 48h. Additionally, the effects of CU and RA at 100 ppm were tested under chronic oral intoxication through continuous insecticide feeding. CU100 significantly reduced mortality in insecticide-exposed bees, except foragers exposed to acetamiprid, while RA showed variable detoxification effects, with RA100 and RA200 improving survival in carbaryl-exposed bees and RA50 enhancing survival of 0.06 µg/bee for newly emerged bees exposed to flupyradifurone. Chronic toxicity assessments confirmed CU100’s superior protective effect over RA100, especially in carbaryl-exposed groups. Gene expression analysis revealed that CU and RA modulated detoxification related genes, enhancing honey bees' resilience by upregulating key detoxification genes in the head and abdomen. These findings suggest that CU and RA offer potential benefits in reducing insecticide toxicity in honey bees. However, further research is needed to assess their effects across different life stages, environmental conditions, and colony dynamics, as well as to elucidate the pathways involved in detoxification gene regulation. A comprehensive understanding of their mechanisms and ecological implications is essential before considering these compounds for practical applications in pollinator health management.

## 1 Introduction

Honey bees are essential pollinators, responsible for fertilizing about one-third of the world’s crop species (1). These insects play a critical role in the pollination of a wide range of fruits, vegetables, and nuts thereby contributing significantly to global agriculture productivity, food security, and the stability of ecosystems. A continued decline in their population could lead to reduced crop yields, higher food prices, and disruptions in natural ecosystems (2, 3). Understanding the factors contributing to their decline is crucial to finding solutions that protect both pollinators and food production systems (4). One major threat to honey bees is pesticide exposure, which can impair their foraging behavior by reducing nectar and pollen collection efficiency and altering navigation and communication within the colony (4). Bees travel up to 10 kilometers from their hives (5), collecting nectar, pollen, and water to sustain their colonies. However, this extensive foraging increases their chances of coming into contact with agrochemicals used in farming. When they return to the hive, they may bring back pesticide-contaminated resources, exposing the entire colony to harmful substances. Over the past few decades, research has repeatedly confirmed the presence of pesticide residues in pollen, beebread, and honey, indicating that honey bee exposure to agricultural chemicals is widespread and persistent (6, 7). This contamination raises concerns about its long-term impact on colony health, reproduction, and survival.

Pesticides are designed to target specific pests, but their toxicity often extends to beneficial insects like honey bees, which share similar metabolic pathways with many pest species (8). The severity of pesticide impact depends on factors such as type, concentration, and exposure duration (9). To mitigate these threats, honey bees rely on natural detoxification mechanisms involving enzyme families like Cytochrome P450 (P450), Glutathione S-transferase (GST), and carboxyl/cholinesterase (CCE) to break down harmful compounds (10, 11). However, their detoxification capacity is limited compared to other insect species, making them particularly vulnerable to environmental stressors, including pesticide exposure (12, 13). Even at sublethal concentrations, honey bees experience decreased survival, reduced foraging efficiency, weakened immune systems, and overall colony decline (14–16).

This lack of detoxification enzymes places honey bees in a vulnerable position, amplifying their susceptibility to pesticide exposure. As modern agricultural practices increasingly rely on chemical pesticides, honey bee populations face heightened risks, contributing to their decline. The inability of honey bees to efficiently metabolize and eliminate toxic substances underscores the urgent need for alternative strategies to enhance their resilience.

One promising approach involves the dietary intake of specific natural compounds that can boost honey bee survival by enhancing their detoxification capacity (17, 18). Studies have demonstrated that phenolic and flavonoid substances, such as p-coumaric acid and quercetin, can improve honey bee survival when exposed to pesticides (17–24). Liao et al. (25) showed the honey bees fed with buckwheat honey which is rich in phenolic acid and flavonoid compounds had higher LD_50_ value for the insecticide bifenthrin in comparison with the bees fed with locust and tupelo honey. Additionally, it has been shown that the consumption of bergamot polyohenolic fraction reduces the toxicity and abnormal behaviour of honey bees intoxicated with deltamethrin (26). This effect is potentially linked to the upregulation of cytochrome P450 enzyme genes, which play a crucial role in detoxification processes (27, 28). These findings suggest that supplementing honey bee diets with bioactive compounds could serve as a practical strategy to enhance their ability to cope with pesticide-induced stress. Curcumin (CU), the active ingredient in the dietary spice turmeric, has been widely studied for its diverse biological activities. With a medicinal history spanning thousands of years, CU is known for its anti-inflammatory, antioxidant, pro-apoptotic, chemopreventive, chemotherapeutic, antiproliferative, wound-healing, and antimalarial properties (29). Although CU is not a naturally occurring component of nectar and pollen, it can be introduced into honey bee diets through supplementation to mimic the role of naturally occurring phytochemicals such as p-coumaric acid and quercetin, which are present in pollen and nectar and are known to contribute to detoxification (30). Studies have shown that honey bee physiology can be influenced by dietary CU. For instance, Farhadi et al. (31) demonstrated that CU supplementation at a dose of 10 mg/L increased body weight and total antioxidant capacity in newly emerged bees, and enhanced longevity under heat stress conditions. Similarly, Rasmussen et al. (32) reported that CU influenced DNA methylation patterns and improved stress resilience in honey bees. These findings suggest that CU supplementation may play a role in modulating honey bee health and resilience to environmental stressors. Similarly, rosmarinic acid (RA), the primary compound in rosemary (*Rosmarinus officinalis*), has been recognized for its antioxidant, anti-inflammatory, anti-allergic, anti-depressive, anti-hyperglycemic, and antimicrobial effects (33). Like CU, RA is not naturally abundant in nectar or pollen, but it represents a phytochemical with potential to complement the effects of naturally occurring compounds. Given their extensive pharmacological benefits, CU and RA may also play a role in enhancing honey bee resilience to pesticide exposure. This study aims to assess their effects on reducing insecticide-induced mortality among worker bees while also evaluating their influence on the expression of cytochrome P450 detoxification genes. By investigating these natural compounds, this research seeks to provide valuable insights into potential dietary interventions that could mitigate insecticide-related threats to honey bee populations, ultimately contributing to their conservation and the sustainability of pollination services in agricultural ecosystems.

## 2 Materials and methods

### 2.1 Honey bee sources and experimental condition

Honey bees (*A. mellifera ligustica*) used in this experiment were collected from the experimental colonies of Andong National University, South Korea. Honey bee colonies containing healthy queens were used as a source of workers. The colonies were free of brood diseases and were not treated to control parasitic mites prior to the experiments because they had very low levels of *V. destructor* infestation (<1%). To obtain newly emerged honey bees, the frames of capped brood from the source hives were incubated overnight at 33 ± 0.5 °C and 70 ± 5% relative humidity (RH) in an incubator, ensuring the emergence of worker bees of the same age. After 24 h, the newly emerged bees were used for the experiments. To obtain 20-day-old honey bees, newly emerged bees were marked, released into their original colonies, and recaptured on day 20. To obtain foragers, honey bees were captured at the entrance of the colony using a sweeping net and kept in the incubator for 24 h to adapt to the experimental conditions and used for the experiment. To ensure consistent environmental conditions in all experiments, the cages were maintained within an incubator set at a constant temperature of 30 ± 1 °C and a relative humidity of 60 ± 10% RH throughout the entire duration of the experiment.

### 2.2 Insecticide

The technical analytical grade (purity > 99%) of flupyradifurone, acetamiprid and carbaryl, were obtained from Sigma-Aldrich (South Korea). All technical analytical grade insecticides were dissolved in acetone to obtain the stock solution and further diluted in different proportions to obtain the specific doses.

### 2.3 Experiment

#### 2.3.1 Evaluate the acute and chronic toxicity of acetamiprid, carbaryl, and flupyrodifrone for honey bees

For contact exposure, both groups of newly emerged and 20-day-old worker bees were exposed on their mesonotum with 2 μL of different doses of one of the insecticides solved in acetone with the aid of a micropipette until 50 bees had been exposed to each treatment. **Table 1** shows the actual doses of each insecticide received by the bees. Control bees received only 2 μL of acetone. After being exposed to insecticide, each group of 10 bees per treatment was placed in a cage (12.7 × 8.5 × 14.5 cm) with a 3 mesh/cm wire screened wall on sides with plastic covering on the top and bottom. The bees intoxicated with each dose of insecticide were fed with 50% (w/v) sugar syrup. Treated bees in the cages were observed until 48h. The number of live and dead bees was recorded at 48h and after treatment.

**Table 1:** Pesticides and the range of applied doses (µg/bee) used to evaluate dose-dependent mortality in newly emerged and 20-day-old honey bees (*Apis mellifera*).

Pesticide	Newly emerged bees (ug/bee)	20-day-old bees (ug/bee)
Acetamiprid	0.06, 0.2, 0.6, 1.8, 5.4	0.1, 1, 10, 50, 100
Caebaryl	0.02, 0.04, 0.06, 0.08, 0.1	0.01, 0.1, 0.25, 0.38, 0.5, 1
Flupyradifurone	0.123, 1.23, 12.3, 122.8, 245.6	0.3, 3, 30, 100, 200

In order to evaluate the chronic toxicity of each insecticide, newly emerged worker bees were provided with different dilutions of the respective insecticide in 50% (w/v) sugar solution. The bees were allowed *ad libitum* access to this solution for a duration of 10 days. The concentrations of each insecticide received by the bees are detailed in **Table 2**. Insecticide doses were selected based on preliminary bioassays that revealed age-dependent differences in sensitivity. To allow meaningful comparisons between age groups, the doses were chosen to produce similar levels of mortality within each group, rather than applying uniform doses across ages. Daily food consumption was recorded for each treatment group, and the average consumption is presented in **Supplementary Table S2**. For the control group, honey bees received only sugar syrup for the same 10-day period, *ad libitum*. Each treatment consisted of groups of 10 bees, and these groups were enclosed in cages measuring 12.7 × 8.5 × 14.5 cm. This experimental design was replicated five times to enhance the reliability of the results. The condition and status of the treated bees in the cages were observed daily over the 10 days.

**Table 2:** Pesticides and the range of applied concentrations (ppm) used to assess concentration-dependent mortality in honey bees (*Apis mellifera*).

Pesticide	Applied concentrations (ppm)
Acetamiprid	10, 25, 50, 100, 150
Carbaryl	0.625, 1.25, 2.5, 5, 10
Flupyradifurone	12.5, 25, 50, 100, 200

#### 2.3.2 Effect of short-term (48h) and long-term feeding by different concentrations of CU and RA on the longevity of honey bees

To evaluate the possible effect of 48h feeding of different concentrations (50, 100, 200 ppm) of CU and RA on the longevity of caged honey bees, the newly emerged honey bees were fed with CU and RA-supplemented sugar syrup for 48h *ad libitum*. The control group only received 50% (w/v) sugar syrup in this period. After 48h the feeders was changed and honey bees in all treatments received only sugar syrup until the last honey bee’s death. This test was conducted in three replications.

To evaluate the effect of long-term CU and RA-supplemented feeding on the longevity of honey bees, 90 newly emerged honey bees were selected for each treatment group in six replications. These bees were then provided with a sugar syrup supplemented with three concentrations (50, 100, and 200 ppm) of the respective compounds (CU or RA) *ad libitum*. This continuous feeding regime extended until the last honey bee in each group reached the end of its natural lifespan. A control group was included in the study to provide a baseline for comparison. The control group exclusively received a sugar syrup of 50% (w/v). This allowed for a clear contrast between the effects of dietary supplementation and the baseline longevity of honey bees.

#### 2.3.3 Detoxification effects of CU and RA on the survivorship of newly-emerged honey bees and foragers, individually intoxicated with insecticides

In order to evaluate the effect of CU or RA-supplemented feeding on reducing honey bee mortality in newly-emerged and foragers intoxicated with lethal dose of acetamiprid, carbaryl and flupyradifurone (**Table 3**), each bee was individually intoxicated topically on their mesonotum with 2 μL of insecticide solved in acetone, then treated with three different concentrations (50, 100 and 200 ppm) of CU and RA *ad libitum*. Control group only received 50% (w/v) sugar syrup. After being exposed to insecticide, each group of 10 bees per treatment was placed in a cage (12.7 × 8.5 × 14.5 cm) with a 3 mesh/cm wire screened wall on sides with plastic covering on the top and bottom. This experiment was conducted with six replications. The mortality of honey bees was recorded after 3, 12, 24 and 48h post treatment.

**Table 3:** Estimated 48-hour contact LD_50_ values (µg/bee) and associated 95% confidence limits (CL), slope ± standard error (SE), intercept, chi-square (χ²), and degrees of freedom (df) obtained from probit analysis for newly emerged and 20-day-old honey bees (*Apis mellifera*) exposed to three insecticides.

Compounds	Probit analysis		95% CL	Slope ± SE	Intercept	χ2	Df
	N	48h-LD_50_ (µg/bee)					
Newly emerged							
Carbaryl	250	0.063	0.043 - 0.089	6.2 ± 0.7	228.0	7.4	13
Flupyradifurone	198	15.6	5.0 - 43.5	1.0 ± 0.1	56.2	3.8	18
Acetamiprid	300	1.039	0.72 - 1.495	1.49 ± 0.14	42.01	4.98	28
20-day-old							
Carbaryl	300	0.513	0.29-1.3	1.23 ± 0.1	8.39.0	4.6	27
Flupyradifurone	250	24.1	11.9-49.6	0.92 ± 0.1	4.3	45.6	23
Acetamiprid	250	15.3	5.5-57.0	0.615 ± 0.1	3.7	51.9	28

#### 2.3.4 Detoxification effects of CU and RA on the survivorship of newly-emerged honey bees, chronically intoxicated with insecticides

In order to study the effect of CU and RA-supplemented feeding on reducing honey bee mortality in newly-emerged honey bees chronically intoxicated orally with lethal concentrations of acetamipirid (50 and 100 ppm), carbaryl (2.5 and 5 ppm), and flupyradifurone (50 and 100 ppm) *ad libitum* over a 10-day period. To assess the potential detoxification effects of CU or RA on insecticides, CU and RA were added to the insecticide-contaminated food at a concentration of 100 ppm. For each insecticide concentration, a control group received contaminated food without phenolic compounds. Additionally, a positive control group was included, receiving only sugar syrup without any insecticide treatment. The honey bees were housed in plastic cages measuring 12.7 × 8.5 × 14.5 cm, featuring circular ventilation openings at the top and bottom for proper airflow. Food was provided through two Eppendorf tubes, each featuring three side holes. The mortality of the intoxicated honey bees was recorded daily.

#### 2.3.5 RNA extraction, real-time PCR and gene expression analysis

To investigate the impact of CU or RA-supplemented feeding on the gene expression profile of worker bees, the newly-emerged bees were provided with CU100, RA50, and RA200 for 24 h in three separate replications. The control group received only a 50% (w/v) sugar syrup. We specifically focused on CU100 for Curcumin due to favorable results in detoxification experiments, while for RA, we encountered challenges in determining the optimal concentration due to varied outcomes across different insecticides. We collected five samples from each replication in every treatment group, storing them in -80 until RNA extraction. Total RNA was isolated from the head and abdomen of pooled five individuals from each replication using the RNeasy Mini Kit (Qiagen, Germany). Subsequently, 1 μg of extracted RNA per treatment was utilized to synthesize complementary DNA (cDNA) using the BioFACT Reverse Transcription Kit (Daejeon, South Korea). The resulting cDNAs were adjusted to 50 μL with sterile water and stored at –80 °C for further analysis. For real-time PCR (RT-PCR), each reaction comprised 100 ng of cDNA from each treatment, 10 pM of gene-specific primers (refer to **Supplementary Table S1**), SYBR green master mix (BioFACT), and nuclease-free water, reaching a final volume of 20 μL. The PCR cycle involved initial denaturation at 95 °C for 15 min (1 cycle), followed by 40 cycles of denaturation at 95 °C for 30 seconds, annealing at 52 °C for 30 seconds, and extension at 72 °C. Fluorescence was measured post-extension, and a dissociation step (95 °C for 15 seconds, 52 °C for 60 seconds, 95 °C for 15 seconds) validated the amplification of a single product in each reaction. RPS5 served as the reference gene. The relative quantities of Catalase (CAT), Superoxide Dismutase 1 (SOD1), and detoxification-related genes, including Cytochrome P450 9Q1 (CYP9Q1), CYP9Q2, CYP9Q3, CYP6S3, CYP6S4, CYP6S10, and Glutathione S-transferase D1 (GSTD1), were determined using threshold cycle (Ct) values and analyzed using the 2^-ΔΔCt method, following established protocols (34). Gene expression levels were normalized against RPS5 (Ribosomal Protein S5), a housekeeping gene. Negative controls excluded cDNA templates, and all reactions were performed in triplicate.

#### 2.3.6 Statistical analysis

Probit analysis was used to determine the lethal doses (LD50) and concentrations (LC50) of each insecticide for honey bees using SPSS version 16. Additionally, Probit analysis was applied to assess the lethal time (LT50) during 48-hour feeding and long-term feeding with supplementary food. Kaplan-Meier survival curves with *post-hoc* comparisons were constructed using the survival package in R version 4.2 to evaluate differences in mortality patterns across treatments during 48-hour feeding and long-term feeding of phenolic compounds. To assess the impact of different concentrations of CU and RA on honey bee survival in the acute toxicity test, the Scheirer-Ray-Hare Test was applied, as the data were not normally distributed based on the Shapiro-Wilk Test. Where significant differences were observed, the Mann-Whitney U test was used to compare treated and control groups. Additionally, Kaplan-Meier survival analysis was used to evaluate the effects of RA100 and CU100 on honey bees chronically exposed to insecticides. For gene expression analysis, one-way ANOVA (p > 0.05) was used to compare the mean relative gene expression in the RA groups, while a t-test was applied for the CU group, as only one concentration (CU100) was used. This comprehensive statistical analysis provided an in-depth evaluation of insecticide effects on honey bee longevity and survival under various conditions.

## 3 Results

### 3.1 Evaluate the acute and chronic toxicity of acetamiprid, carbaryl, and flupyrodifrone for honey bees.

Different doses of insecticides (acetamiprid, carbaryl, and flupyradifurone) were administered to assess mortality levels in intoxicated bees. Probit analysis of dose-dependent mortality in honey bees revealed that the LD_50_ value of acetamiprid for newly emerged honey bees was 1.039 µg/bee, whereas for 20-day-old bees, it was 15.3 µg/bee. For carbaryl, the LD_50_ value was 0.063 µg/bee for newly emerged honey bees and 0.513 µg/bee for 20-day-old bees. In the case of flupyradifurone, Probit analysis indicated an LD_50_ value of 15.6 µg/bee for newly emerged honey bees, while for 20-day-old bees, the LD_50_ value was 24.1 µg/bee (**Table 3**).

2- Chronic toxicity of flupyrodifrone, acetamiprid and carbaryl on worker honey bees.

In this study, varying concentrations of three insecticides, acetamiprid, carbaryl, and flupyradifurone, were administered to assess their effects on honey bee mortality. For acetamiprid, the results indicated a dose-dependent increase in mortality among newly emerged honey bees. Specifically, the three lower concentrations of 10, 25 and 50 ppm led to a gradual increase in mortality, with an approximate rise of 20 percent in mortality compared to the control group, which solely received sugar syrup. At 100 ppm, approximately 70 percent of the honey bees succumbed to mortality, and this figure soared to a staggering 90 percent at 150 ppm (as shown in **Supplementary Figure S2**). The calculated LC_50_ was 45.7 ppm. In the case of carbaryl, a comparable pattern was observed. At the two lower concentrations, 0.625 ppm and 1.25 ppm, there was an approximately 15 percent mortality rate. However, with an increase in concentration to 2.5, 5 and 10 ppm, the mortality rate rose to 35, 75 and 100 percent respectively. The estimated LC_50_ of carbaryl was 2.61 ppm. In the case of flupyradifurone, the two lower concentrations, 12.5 and 25 ppm, yielded approximately 15–20 percent mortality. However, at a concentration of 50 ppm, there was an observed mortality rate of about 50 percent. The mortality of honey bees escalated to over 90 percent when exposed to 200 ppm, with all honey bees succumbing within five days in the group that received 200 ppm of flupyradifurone. The calculated LC_50_ was 30.56 ppm (**Table 4**).

**Table 4:** Estimated 10-day oral LC_50_ values (ppm) and associated 95% confidence limits (CL), slope ± standard error (SE), intercept, chi-square (χ²), and degrees of freedom (df) obtained from probit analysis for honey bees (*Apis mellifera*) exposed to three insecticides.

Compounds	Probit analysis		95% CL	Slope ± SE	Intercept	χ2	Df
	N	Estimated toxicity (ppm)					
Acetamiprid	250	45.73	35.47-59	1.97 ± 0.21	-3.27	44.19	28
Carbaryl	250	2.61	2.16-3.17	2.86± 0.26	-1.19	42.05	28
Flupyradifurone	250	30.56	26.2-35.39	2.86± 0.29	-4.24	25.17	28

### 3.2 Effect of short-term (48h) and long-term feeding by different concentrations of CU and RA on the longevity of honey bees

**Figure 1:**
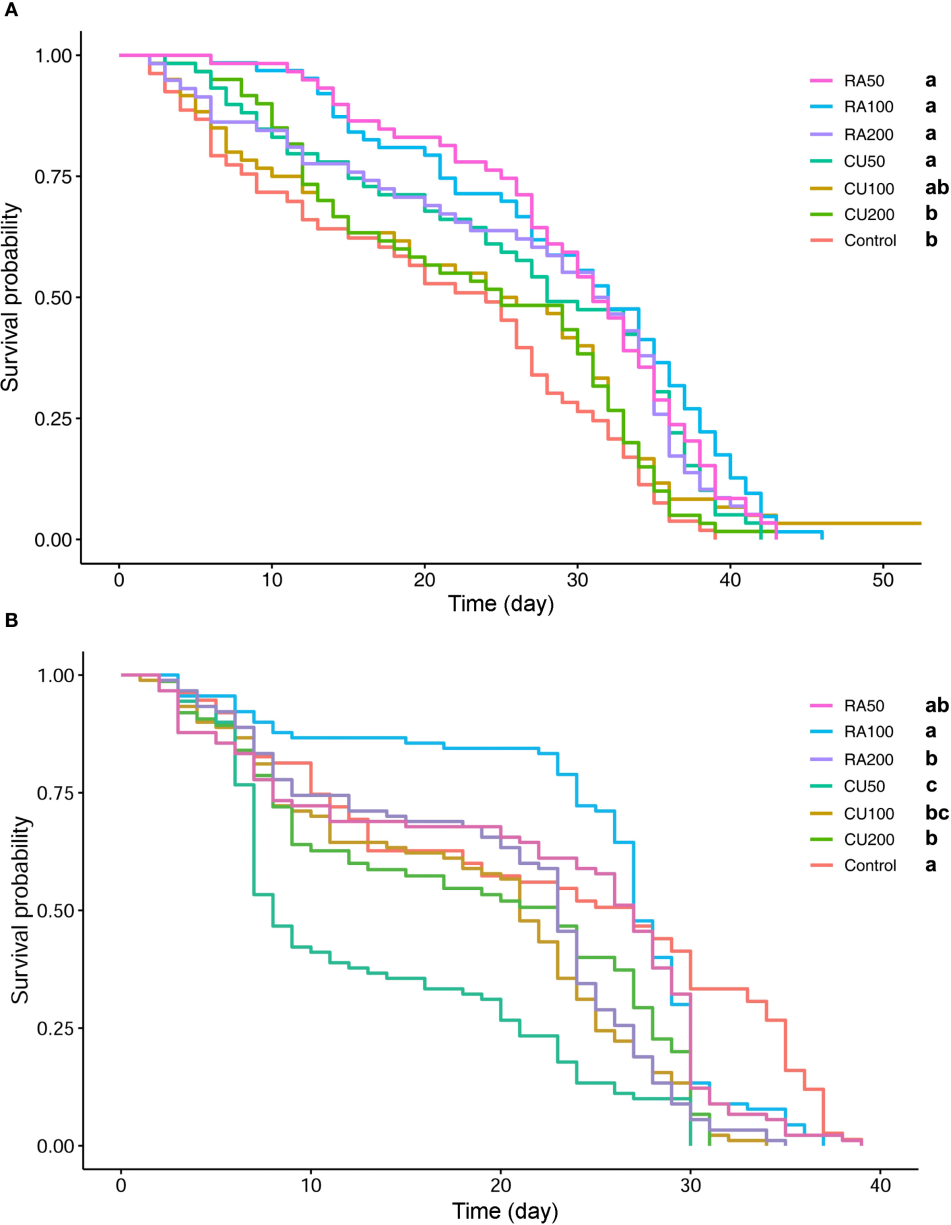
Kaplan–Meier survival curves of honey bees fed with different concentrations of curcumin (CU) and rosmarinic acid (RA) for 48 hours **(A)** and throughout their lifespan **(B)**. Treatments included RA at 50, 100, and 200 ppm (RA50, RA100, RA200) and CU at 50, 100, and 200 ppm (CU50, CU100, CU200), compared with an untreated control group. Different letters indicate statistically significant differences among treatments (log-rank test, *P* < 0.05).

This experiment was conducted to evaluate the possible effect of short-time and long-time feeding of CU and RA supplementary food on honey bee health and survival. In the 48h feeding test, the difference in cage bee longevity among treatments was significant (**Figure 1A**). The longest survival was found in bees fed RA100 and RA50 followed by bees fed RA200, CU50. The lowest lifespan was observed in the control group and a group of workers fed with CU200 (**Figure 1A**; **Supplementary Table S2**).

In the long-term feeding experiment, the Kaplan-Meier survival analysis of caged bees revealed noteworthy variations in longevity among the treatment groups. Within the RA-treated groups, RA50 and RA100 exhibited no adverse effects on the survival of treated honey bees. However, long-term exposure to RA200 supplement significantly decreased honey bee survival compared to the control group. The estimated LT_50_ for the control group was 532.8 h, whereas for RA-treated groups, it was 481.44h, 534h, and 415.2h for RA50, RA100, and RA200, respectively. The higher LT_50_ value for RA100 was attributed to improved early-stage survival, but mortality increased after three weeks, resulting in comparable survival rates between RA100 and the control group. However, all CU-treated groups exhibited significantly lower survival than the control group. Honey bees treated with CU50 experienced high early-stage mortality, leading to significantly lower survival probability compared to CU100 and CU200. The estimated LT_50_ values were 379.2h and 364.8h for the CU100 and CU200 groups, while the LT_50_ for the CU50 group was notably lower at 252h (**Figure 1B**).

### 3.3 Detoxification effects of CU and RA on the survivorship of newly emerged honey bees individually intoxicated with insecticides

**Figure 2:**
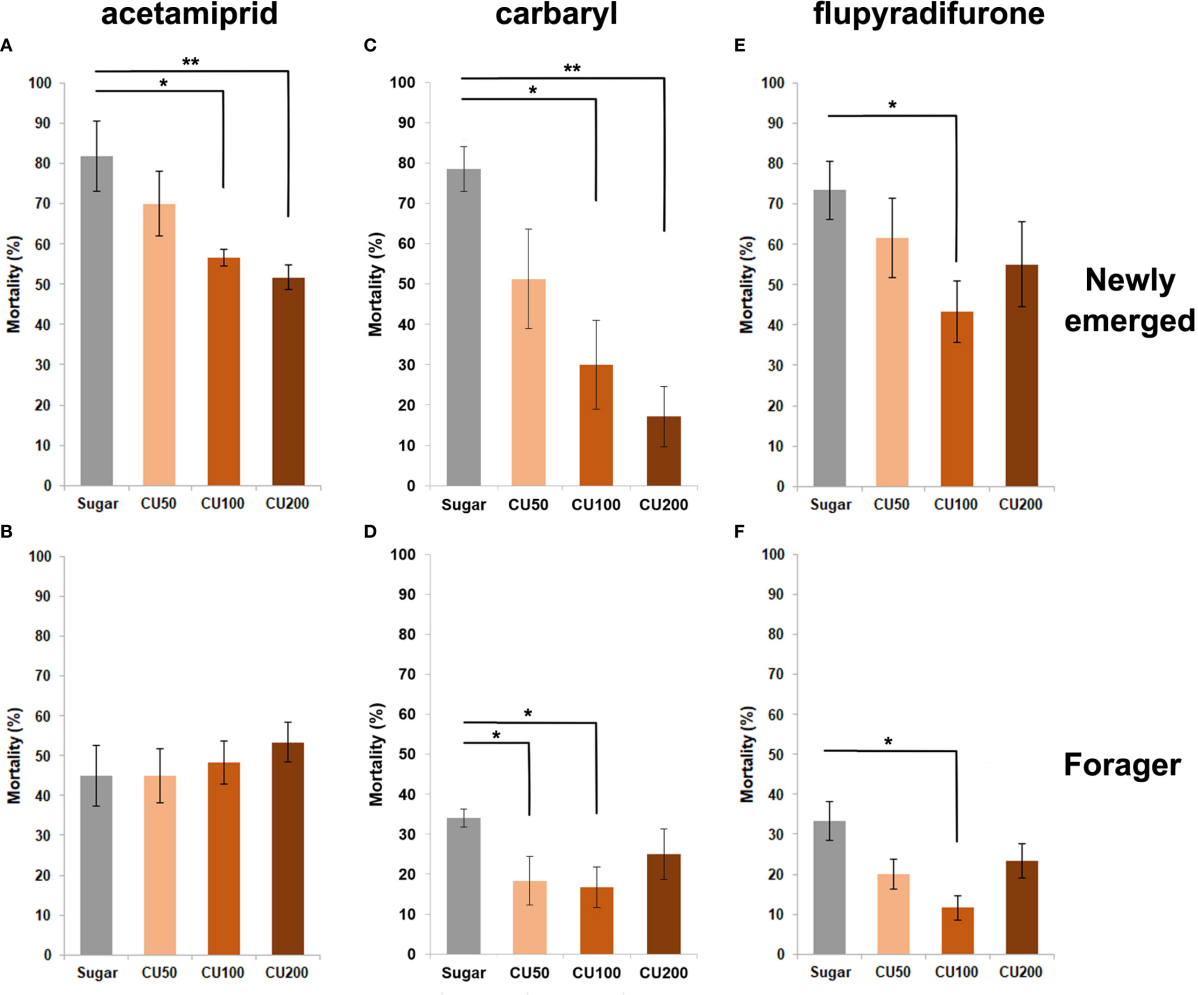
Effect of different concentrations (50, 100, and 200 ppm) of curcumin (CU) on the mortality of newly emerged bees **(A, C, E)** and foragers **(B, D, F)** exposed to acetamiprid **(A, B)**, carbaryl **(C, D)**, and flupyradifurone **(E, F)**. Bees were orally fed CU-supplemented food for 48 h following topical exposure to the pesticides. Mortality was assessed after 48 h. Bars represent mean ± SE. Statistical comparisons were performed using one-way ANOVA followed by Tukey’s HSD *post-hoc* test. Asterisks indicate significant differences compared to the sugar-fed control group: * p < 0.05, ** p < 0.01.

**Figure 3:**
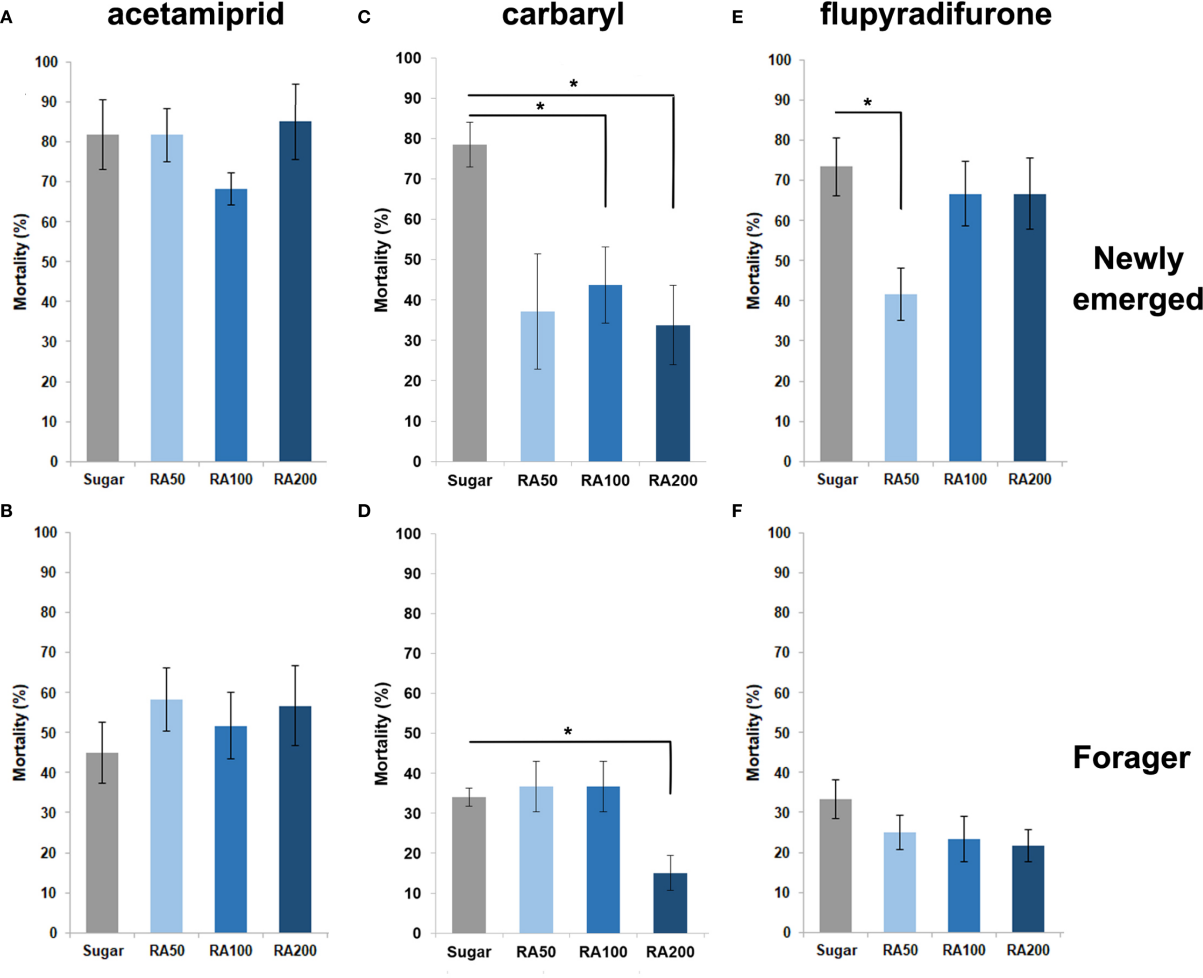
Effect of different concentrations (50, 100, and 200 ppm) of rosmarinic acid (RA) on the mortality of newly emerged bees **(A, C, E)** and foragers **(B, D, F)** exposed to acetamiprid **(A, B)**, carbaryl **(C, D)**, and flupyradifurone **(E, F)**. Bees were orally fed CU-supplemented food for 48 h following topical exposure to the pesticides. Mortality was assessed after 48 h. Bars represent mean ± SE. Statistical comparisons were performed using one-way ANOVA followed by Tukey’s HSD *post-hoc* test. Asterisks indicate significant differences compared to the sugar-fed control group: *p < 0.05.

This experiment was conducted to evaluate the effect of using different concentrations of CU and RA to reduce the mortality of intoxicated honey bees with the lethal doses of three insecticides, acetamiprid, carbaryl and flupyradifurone. The results showed that post-feeding of different concentrations of CU and RA decreased the mortality of honey bees intoxicated with flupyradifurone but the difference was only significant when workers treated with RA50 (W = 3, p-value = 0.019) and CU100 (W = 7.5, p-value = 0.0104) (**Figures 2E**, **3E**) and foragers treated with CU100 (W = 2, p-value = 0.0108) (**Figures 2F**, **3F**). In the case of carbaryl, post-feeding of all concentrations of CU and RA decreased the mortality of nurse bees but the difference was significant when nurses were treated with RA100 (W = 8.5, p-value = 0.026), RA200 (W = 5.5, p-value = 0.0104), CU100 (W = 6.5, p-value = 0.0141), and CU200 (W = 1, p-value = 0.0027) (**Figures 2C**, **3C**). Foragers treated with RA200 (W = 1.5, p-value = 0.014), and CU100 (W = 3, p-value = 0.026) had significantly lower mortality in comparison with the control group (**Figures 2D**, **3D**). In case of acetamiprid, post-feeding of CU100 (W = 5, p-value = 0.038) and CU200 (W = 3.5, p-value = 0.021) decreased the mortality of nurse bees significantly compared to the control group (**Figures 2A**, **3A**), however, the difference between the mortality of foragers was not significant (**Figure 2B**, **3B**).

### 3.4 Detoxification effects of CU and RA on the survivorship of newly emerged honey bees, chronically intoxicated with insecticides

**Figure 4:**
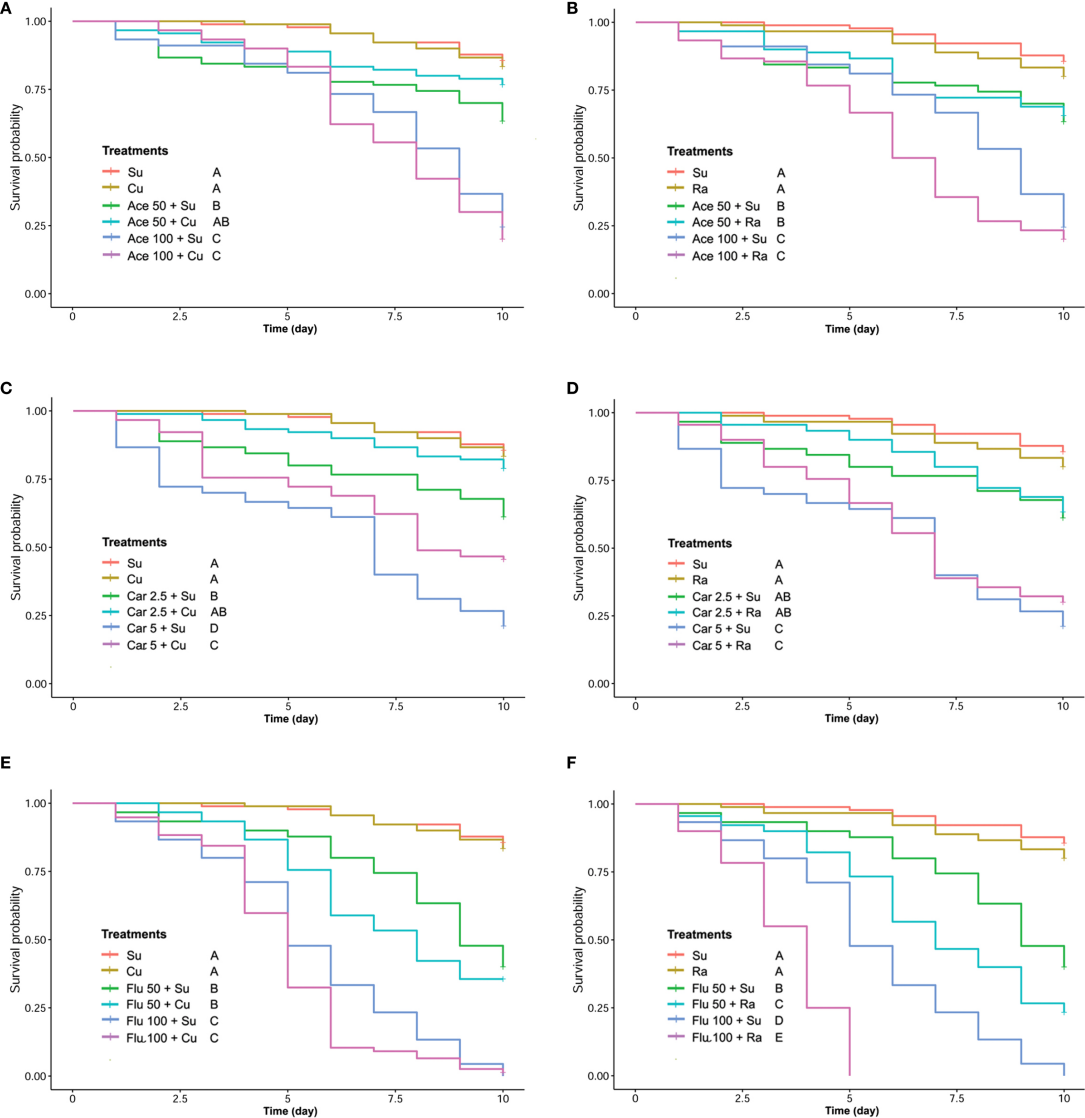
The Kaplan-Meier survival analysis of honey bees chronically exposed to different concentrations of acetamiprid **(A, B)**, carbaryl **(C, D)** and flupyradifurone **(E, F)** while providing CU100 **(A, C, E)** or RA100 **(B, D, F)**.

The detoxification effect of phenolic compounds on honey bees chronically intoxicated with two concentrations of insecticides was also evaluated. The control groups received only insecticide while the treatment group received insecticide with 100 ppm of phenolic compound (CU or RA). Positive control only received sugar syrup. In case of carbaryl, the 10-day survival analysis revealed a mitigating effect of CU on insecticide-induced harm in both lower and higher concentrations. Conversely, in the RA-fed group, the results presented a contrasting picture. There was no significant difference observed between the insecticide-treated group and the group treated simultaneously with insecticide and RA. Although honey bee mortality was lower in the RA group with the higher concentration of carbaryl, the difference was not statistically significant (**Figures 4C, D**).

In the flupyradifurone experiment, the results indicated that both CU and RA were ineffective in reducing the mortality of honey bees exposed to long-term concentrations of flupyradifurone, both in lower and higher concentrations. Surprisingly, RA supplementation significantly increased honey bee mortality in both lower and higher concentrations (**Figures 4E, F**). In honey bees exposed to acetamiprid, the 10-day survival analysis revealed a mitigating effect of CU on insecticide-induced harm in lower concentration of acetamiprid. The application of long-term feeding of CU was not effective in decreasing the mortality of honey bees intoxicated with higher concentration of acetamiprid. Conversely, in the RA-fed group, the results demonstrated that there was no significant difference observed between the insecticide-treated group and the group treated simultaneously with insecticide and RA (**Figures 4A, B**).

We observed a statistically significant increase in food consumption among the group treated with acetamiprid. Our findings suggest that this heightened consumption can be linked to distinctive signs of toxicity observed in honey bees exposed to acetamiprid. It appears that honey bees affected by acetamiprid exhibit symptoms of food intake disorder, leading them to disperse the solution within the cage rather than consuming it. Consequently, droplets of the solution accumulate at the bottom of the cage. For this reason, we changed the bottom of the cages in all treatment goups daily (**Supplementary Figure S4B**).

### 3.5 Gene expression analysis

**Figure 5:**
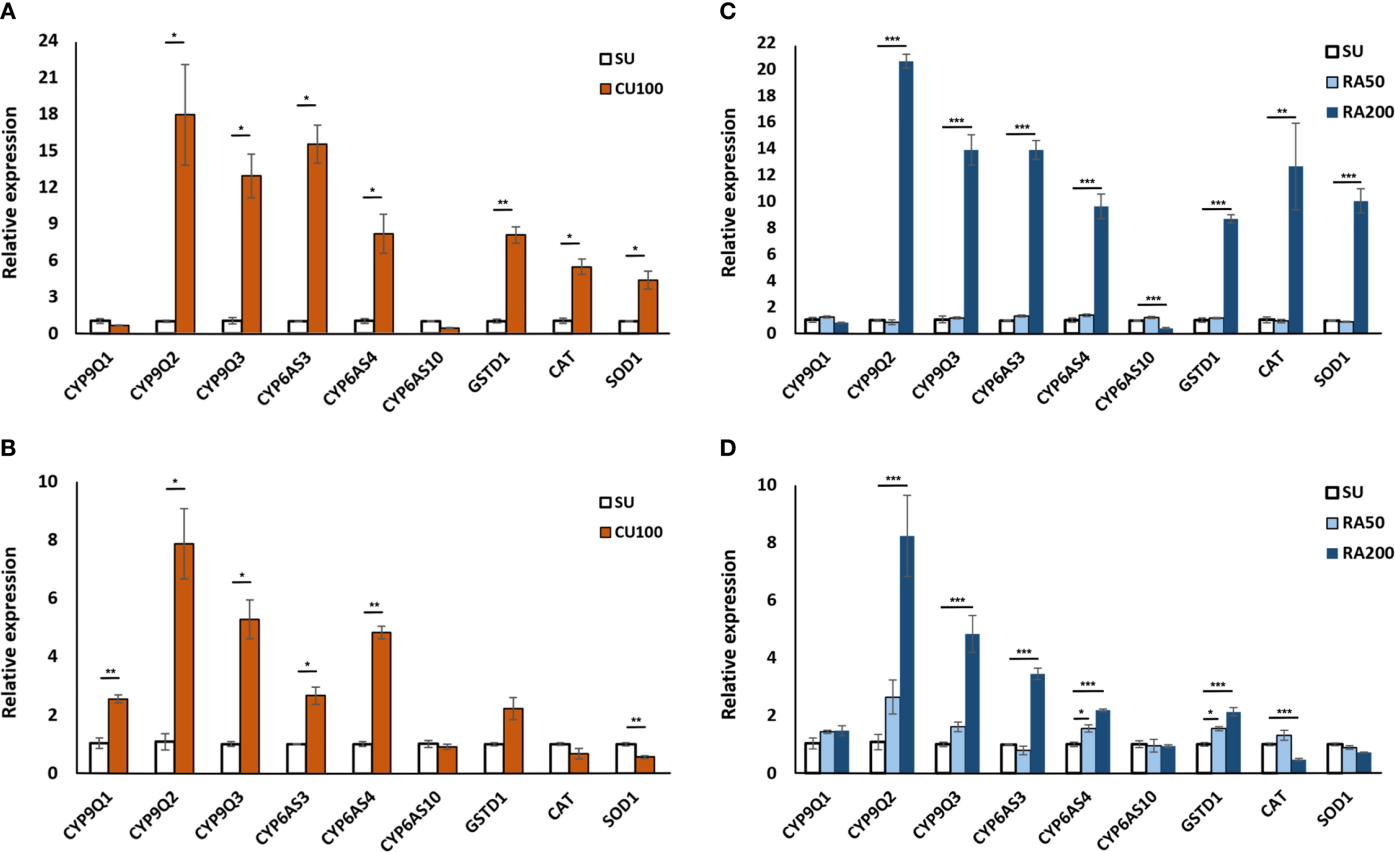
Gene expression profiles in the head **(A, C)** and abdomen **(B, D)** of honey bees at 24 hours’ post-treatment. **(A, B)** show bees fed with CU100, while **(C, D)** show bees fed with two concentrations of rosmarinic acid (RA50 and RA200). SU represents the control group fed only sugar syrup. Relative expression levels of detoxification- and antioxidant-related genes were measured. Asterisks indicate statistically significant differences compared to the control group (*p < 0.05, ** p < 0.01, ***p < 0.001).

Gene expression analysis was conducted to delve into the potential impact of CU and RA on detoxification-related genes. Overall, a consistent pattern emerged regarding the expression of detoxification and stress-related enzymes when honey bees were exposed to CU and RA compounds, with a few exceptions. CU100 notably boosted the expression of CYP9Q2, CYP9Q3, CYP6AS3, CYP6AS4, and GSTD1 in the head, yet it did not show a similar effect on CYP9Q1 and CYP6AS10. A parallel pattern was observed in the gene expression profile analysis of detoxification-related genes in the abdomen, except for CYP9Q1, where an increase in expression was noted in the CU100 group (**Figures 5A, B**). In contrast, the RA50 treatment for worker bees led to higher expression only in CYP6AS4 in the abdomen compared to the control group. However, like CU100, the application of higher concentrations of RA (RA200) enhanced the expression of CYP9Q2, CYP9Q3, CYP6AS3, CYP6AS4, and GSTD1 in both the head and abdomen. Interestingly, CU and RA in all concentrations did not enhance the expression of CYP6AS10, and its expression even decreased significantly in the head with RA200 treatment (**Figures 5C, D**).

Furthermore, the application of CU100, and RA200 increased the expression of CAT and SOD1 in the head but decreased their expression in the abdomen, although these differences were not always statistically significant compared to the control group (**Figure 5**).

## 4 Discussion

Our study provides a comprehensive assessment of the potential of CU and RA to mitigate the insecticide-induced harm in honey bees. Our findings underscore the promising detoxification capabilities of CU and RA, significantly enhancing honey bee survival post-exposure. Both compounds, particularly at specific concentrations, effectively reduce mortality across different insecticide classes. It is important to note that the pesticides used in this study, acetamiprid, carbaryl, and flupyradifurone, act via distinct mechanisms. Acetamiprid is a neonicotinoid that targets nicotinic acetylcholine receptors, disrupting neuronal signaling (35). Carbaryl, an organophosphate, inhibits acetylcholinesterase, leading to accumulation of acetylcholine and overstimulation of neurons (36). Flupyradifurone, a butenolide, also targets nicotinic receptors but with a different binding profile compared to neonicotinoids (37). These mechanistic differences can influence both the pattern of toxicity and the detoxification pathways induced in honey bees.

In acute toxicity tests, CU100 significantly reduced mortality in all insecticide-exposed honey bees, except for foragers exposed to acetamiprid (**Figure 2**), This difference may reflect age- and task-related variations in detoxification capacity between nurse bees and foragers. RA also reduced mortality in carbaryl-exposed bees but was less effective against flupyradifurone and acetamiprid at lower concentrations (**Figure 3**). These findings indicate that detoxification responses vary depending on both the insecticide and the physiological state or role of the honey bee, consistent with previous observations that honey bee detoxification enzyme activity, including cytochrome P450 monooxygenases, can change with age and task allocation (38). Liao et al. (20) further highlighted the challenges in optimizing dietary concentrations of p-coumaric acid and quercetin in reducing insecticide toxicity, emphasizing the complex interactions between phytochemicals and pesticides in honey bee diets.

In our chronic toxicity assessment, CU100 performed better than RA100 in reducing mortality among carbaryl-exposed bees (**Figure 4**). However, RA100 did not consistently enhance survival and even increased mortality in flupyradifurone-exposed bees (**Figure 4F**). This outcome may be explained by the physiological stress associated with increased intake of RA in combination with insecticide exposure (**Supplementary Figure S4F**), potentially leading to metabolic overload or disruption of detoxification pathways. Honey bee detoxification relies on enzymes such as cytochrome P450s, glutathione-S-transferases, and carboxylesterases (10, 12), which can be differentially induced or inhibited depending on both the insecticide and dietary compound (17, 38). The increased consumption of RA alongside the insecticide may have surpassed the optimal range for beneficial detoxification effects, thereby exacerbating toxicity.

We also examined the impact of short-term (48h) and long-term feeding of different concentrations of CU and RA on honey bee longevity. In the short-term feeding test, the results showed that these compounds not only had no adverse effects on worker bee longevity, but also that all concentrations of RA (specifically 50 and 100 ppm) and CU50 significantly improved honey bee longevity. Such beneficial effects are consistent with previous studies showing that dietary flavonoids and phenolic compounds, including p-coumaric acid and quercetin, can increase bee survival under pesticide or environmental stress (17, 19, 20). The negative impact of lifelong feeding with most concentrations of CU and RA, particularly the pronounced effect of CU50, indicates that chronic supplementation may overstimulate detoxification and metabolic pathways, potentially leading to physiological stress or resource allocation trade-offs that reduce longevity. Similar dose- and duration-dependent effects have been reported for phenolic compounds, where prolonged exposure can produce synergistic toxicity in combination with certain pesticides (20, 23). These findings underscore importance of optimizing both the concentration and duration of phytochemical supplementation in honey bee diets to maximize protective effects while avoiding potential long-term adverse consequences.

Gene expression analysis provided insights into detoxification and stress responses in bees treated with CU and RA. Metabolic detoxification, involving cytochrome P450 monooxygenases (CYPs), carboxylesterases (COEs), and glutathione S-transferases (GSTs), plays a crucial role in resisting pesticides (10).

The distinct expression patterns observed suggest that these compounds can differentially modulate detoxification-related genes, potentially enhancing the honey bees’ resilience to pesticide exposure. CU100 upregulated several CYP genes (CYP9Q2, CYP9Q3, CYP6AS3, CYP6AS4, and GSTD1) in the head. These genes are involved in phase I and phase II detoxification pathways: CYP9Q and CYP6AS subfamily enzymes participate in oxidative metabolism of xenobiotics (phase I), while GSTD1 is critical for conjugation and detoxification reactions (phase II) (10, 19, 27). However, the absence of a similar upregulation in CYP9Q1 and CYP6AS10 suggests a selective effect of CU100 on specific oxidative metabolism pathways. Interestingly, in the abdomen, the expression of CYP9Q1 was increased, indicating tissue-specific responses to CU100 treatment. Previous studies have shown that CYP6AS subfamily enzymes and CYP9Q3 are responsible for metabolizing quercetin and are induced by p-coumaric acid (17, 19, 28). Furthermore, CYP9Q enzymes play a crucial role in detoxifying multiple classes of insecticides, including pyrethroids, organophosphates (27), the N-cyanoamidine neonicotinoids (39), and the anthranilic diamide (40).

RA50 upregulated CYP6AS4 in the abdomen, while RA200 enhanced CYP9Q2, CYP9Q3, CYP6AS3, CYP6AS4, and GSTD1, indicating a dose-dependent effect of RA on detoxification genes, potentially amplifying the detoxification capacity of honey bees at higher concentrations. However, RA200 significantly decreased the expression of CYP6AS10 in the head, which might indicate a complex regulatory mechanism where RA200 downregulates certain detoxification genes while upregulating others. The complexity of detoxification mechanisms has been demonstrated in previous studies analyzing the pathways involved in the detoxification of insecticides. While studies demonstrated the possible role of CYP9Q3 in detoxification of carbaryl (41), honeybee larvae demonstrated elevated levels of expression of CYP9Q2 after being exposed to acetamiprid (42). Additionally, the upregulation of both CYP9Q2 and CYP9Q3 in foragers exposed to flupyradifurone is documented (43). This intricate regulatory balance underscores the complexity of the detoxification response and highlights the selective modulation of specific CYP genes by different compounds.

Furthermore, both CU100 and RA200 treatments increased the expression of catalase (CAT) and superoxide dismutase (SOD1) in the head but decreased their expression in the abdomen (**Figure 4**). This differential expression pattern suggests that while CU and RA can enhance the antioxidant defense in the head, they may not have the same effect in the abdomen, or the mechanisms of antioxidant regulation might differ between these tissues. The increase in CAT and SOD1 in the head is particularly important because neural tissues are highly sensitive to oxidative stress, and reduced antioxidant defense has been associated with cognitive impairments in bees exposed to pesticides. For example, García et al. (44) demonstrated that pesticide exposure induces learning and motor impairments in honey bees, while phytochemicals can mitigate these effects. Likewise, chronic imidacloprid exposure was shown to decrease SOD1 expression in the bee brain, correlating with impaired optomotor (vision-based) behavior (45). Similar downregulation of CAT and SOD has been reported in larvae and pupae exposed to neonicotinoids, leading to increased oxidative stress and potential neural damage (46). Taken together, these findings suggest that the upregulation of CAT and SOD1 in the head observed in our study may contribute to protecting critical neural functions, such as learning, memory, and sensory processing, from pesticide-induced oxidative damage. It is important to note that the concentrations of insecticides used in our laboratory experiments exceed typical field-reported exposures in nectar and pollen. These higher doses were deliberately chosen to allow clear detection of detoxification effects of CU and RA on honey bee mortality and gene expression. Field-reported pesticide residues are generally sublethal and may produce minimal mortality, making it difficult to observe measurable protective effects in short-term assays. Nonetheless, our results provide proof-of-concept evidence that phytochemical supplementation can enhance antioxidant and detoxification responses. Future studies should assess the effects of CU, RA, or naturally occurring phytochemicals at field-realistic doses to confirm ecological relevance for honey bee health in agricultural landscapes.

Although CU100 and RA50 or RA200 were helpful in reducing insecticide-induced mortality and oxidative stress in honey bees, long-term application of these phytochemicals in the honey bee diet decreased the survival of honey bees significantly. This could be due to the continuous enhancement of the expression of detoxification enzymes and other unknown manipulations of honey bee physiology, which can be harmful in long-term scenarios. The adverse effects of continuous feeding on phenolic compounds, such as p-coumaric acid and quercetin, have been previously demonstrated, as these compounds exhibited a synergistic effect on bees exposed to chlorantraniliprole or a combination of propiconazole and chlorantraniliprole (20). Additionally, while phenolic compounds reduced mortality in honey bees exposed to lower concentrations of thiamethoxam, they exhibited a synergistic effect on in-hive-aged honey bees exposed to higher doses of thiamethoxam (23), further emphasizing the potential risks associated with prolonged exposure to these compounds.

While our findings and previous studies indicate the promising potential of phenolic compounds in enhancing honey bee longevity and reducing insecticide-related harm, further research is necessary to fully understand the implications and optimize their use. Specifically, more studies are required to investigate the effects of these compounds on the various life stages of honey bees, including the immature stages. Additionally, it is crucial to conduct both laboratory and field studies to assess the long-term effects and practical applications of these compounds in real-world beekeeping and agricultural settings. Laboratory studies can provide controlled conditions to dissect the precise mechanisms by which these compounds exert their protective effects, while field studies can offer insights into their efficacy and safety in natural environments where bees are exposed to multiple stressors, including pesticides, pathogens, parasites (e.g., *Varroa destructor*, *Nosema* spp.), and nutritional deficiencies (47, 48).

Overall, while CU and RA show great promise in mitigating insecticide-induced harm and enhancing honey bee health, comprehensive and multifaceted research is essential. By exploring the effects of these compounds across different life stages, environmental conditions, and colony dynamics, we can develop a robust understanding of their potential benefits and limitations. This will ultimately contribute to the sustainable management of honey bee populations, which are vital for pollination and agricultural productivity.

## Data Availability

The datasets presented in this study can be found in online repositories. The names of the repository/repositories and accession number(s) can be found in the article/supplementary material.
