# Supplementary material for: Exploring curcumin and rosmarinic acid as potential antidotes for pesticide-induced harm to honey bees
Source: Front Insect Sci. 2025 Oct 22;5:1673140. doi: 10.3389/finsc.2025.1673140 (PMC12586108; doi:10.3389/finsc.2025.1673140)
Supplement: Supplementary file 1 [file Supplementaryfile1.docx]

Table S1: Primers used in our study.

| **Symbol** | **Full gene name** | **Accession no.** | **F/R** | **Primer sequence** | **Mer** | **GC(%)** | **Tm(℃)** | **Amplicon size** | **Reference** |
| --- | --- | --- | --- | --- | --- | --- | --- | --- | --- |
| **RPS5** | 40S ribosomal protein S5 | XM_006570237 | F | GATGTTTCTCCGTTACGACGAGT | 23 | 48 | 60.9 | 114 | Jeon et al., 2020 |
|  |  |  | R | GAGTTCATCGGCTAAACATTCGG | 23 | 48 | 60.3 |  |  |
| **CAT** | Catalase | NM_001178069 | F | CTTGGCCCAAACAATCTGCAAT | 22 | 45 | 60.6 | 151 | Gizaw et al. 2020 |
|  |  |  | R | GACATTCTCTAGGCCCACCA | 20 | 55 | 62.3 |  |  |
| **SOD1** | Superoxide dismutase 1 | NM_001178027 | F | GCGTTCTTCAGGGTGAAGTC | 20 | 55 | 61.2 | 203 | Gizaw et al. 2020 |
|  |  |  | R | ATCAGGTCCACCATGATCCTTT | 22 | 45 | 61.2 |  |  |
| **CYP9Q1** | Cytochrome P450 9Q1 | XM_006562301 | F | ACCTGTCCACGAGGAATCAC | 20 | 55 | 62.5 | 234 | Gizaw et al. 2020 |
|  |  |  | R | CCTTCACCCCGATCGTCTTT | 20 | 55 | 63.2 |  |  |
| **CYP9Q2** | Cytochrome P450 9Q2 | XM_392000 | F | GTTGTTCATCATGGACGGCG | 20 | 55 | 61.5 | 151 | This study |
|  |  |  | R | TGTCCTTCTCGGGCAGTTT | 19 | 53 | 61.9 |  |  |
| **CYP9Q3** | Cytochrome P450 9Q3 | XM_006562300 | F | TACGTGGGCATTTACGAGTTCA | 22 | 45 | 60.2 | 249 | Gizaw et al. 2020 |
|  |  |  | R | CTCGGTCATCAGCTTGAACATA | 22 | 45 | 58.6 |  |  |
| **CYP6AS3** | Cytochrome P450 6AS3 | XM_026444747.1 | F | TCGAAAGGGACGAGGATATG | 20 | 50 | 59.4 | 129 | De Smet et al. (2017) |
|  |  |  | R | AGTCATGGGATGCCTACTGG | 20 | 55 | 62 |  |  |
| **CYP6AS4** | Cytochrome P450 6AS4 | XM_395671.6 | F | CCTGATATTGGAATGCGCCG | 20 | 55 | 61 | 132 | This study |
|  |  |  | R | GATTCCGAAAGCGCAACTCC | 20 | 55 | 61.4 |  |  |
| **CYP6AS10** | Cytochrome P450 6AS10 | XM_016915831.2 | F | CCTGGACCCAAGCCAATAC | 19 | 58 | 61.9 | 153 | This study |
|  |  |  | R | TTATAGCCAGAACACGCACG | 20 | 50 | 58.5 |  |  |
| **GSTD1** | Glutathione S-transferase D1 | NM_001178028.1 | F | GGTGTGGAGAGATAGTGCGA | 20 | 55 | 61.3 | 120 | This study |
|  |  |  | R | TTTCAACGGGGACGCAAATG | 20 | 50 | 61.3 |  |  |


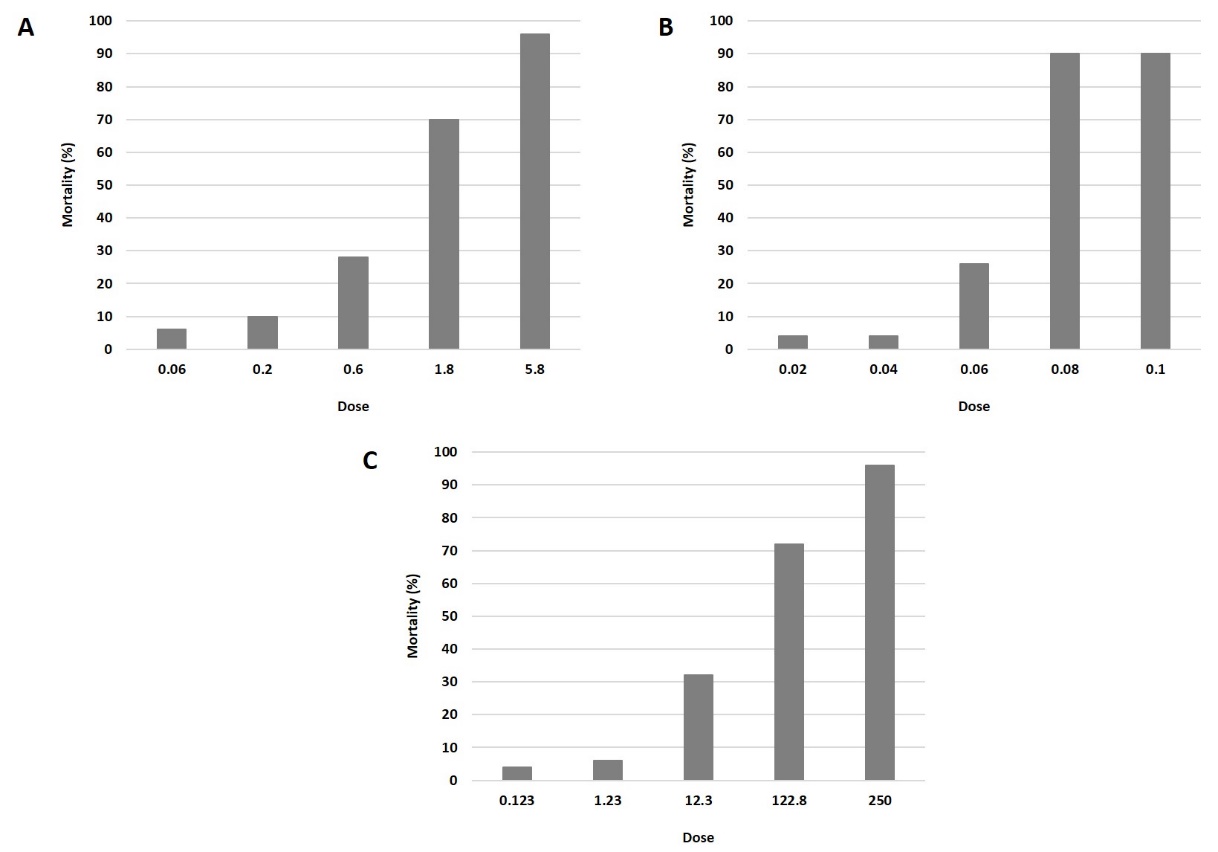


Figure S1- Dose-dependent mortality of newly-emerged honeybees intoxicated with different dosage of acetamiprid (A), carbaryl (B), and flupyradifurone (C).


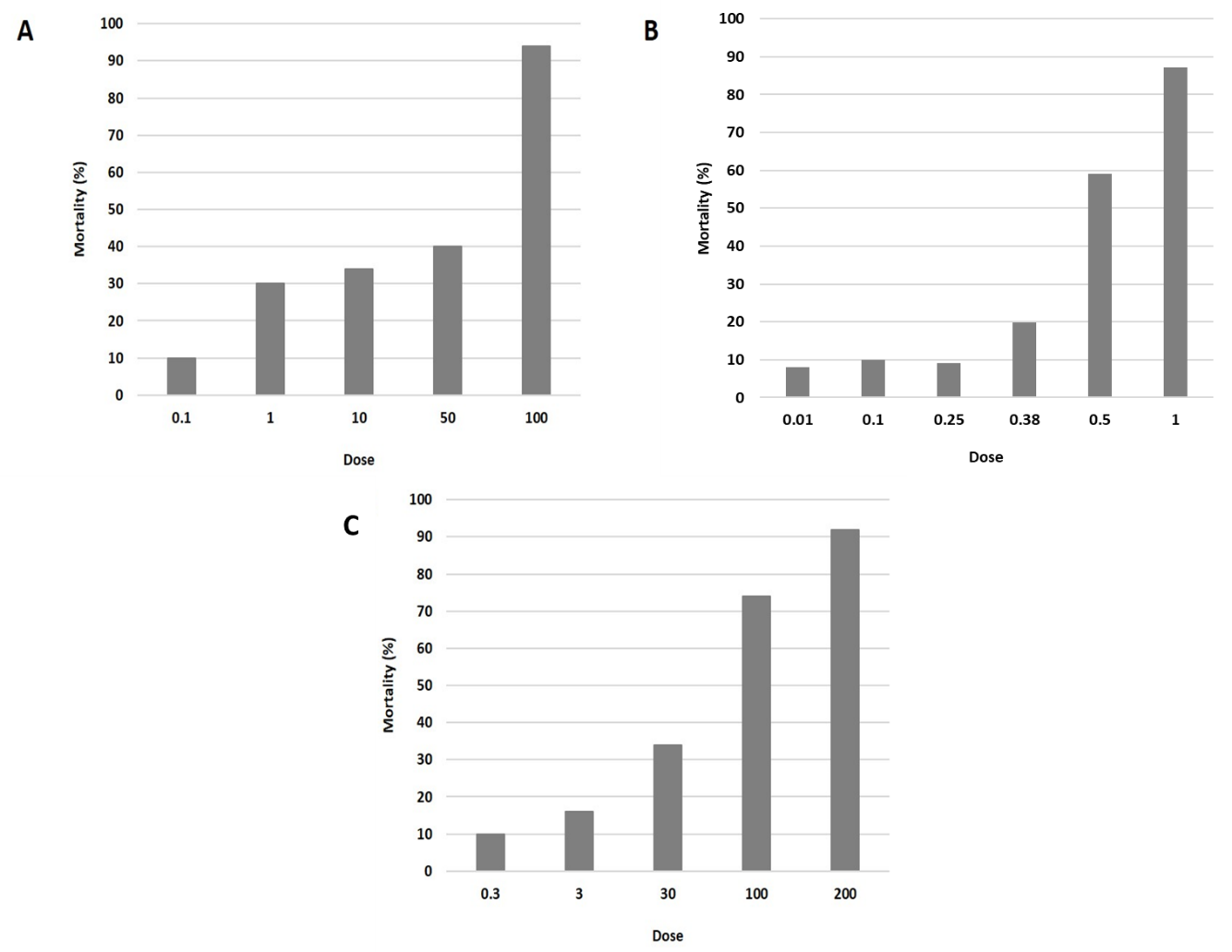


Figure S2 - Dose-dependent mortality of 20-day-old honeybees intoxicated with different dosage of acetamiprid (A), carbaryl (B), and flupyradifurone (C)..


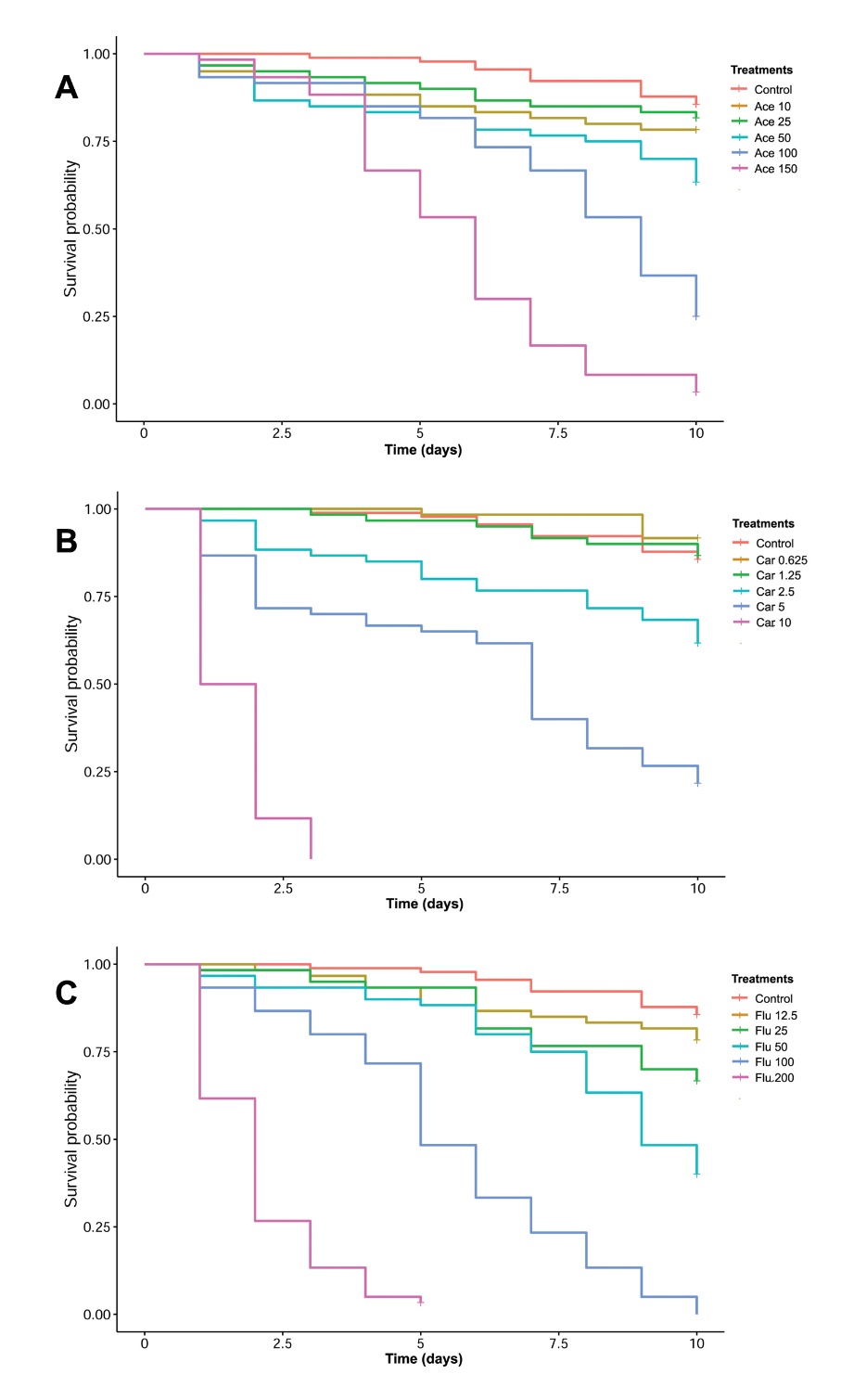


Figure S3- The 10 days Kaplan-Meier survival analysis of honeybees chronically exposed to different concentrations of acetamiprid (A), carbaryl (B) and flupyradifurone (C).

Table S2- Average daily food consumption (µL/bee ± SEM) and corresponding pesticide intake (µg/bee) across different treatments. Pesticide intake values were calculated based on the provided concentrations (ppm) and mean daily consumption.

| Treatment | Consumption  (µL/bee/day, mean ± SEM) | Pesticide consumption  (µg/bee/day, mean ± SEM) |
| --- | --- | --- |
| Ace 10 | 18.56 ± 2.1 | 0.185581 ± 0.020 |
| Ace 20 | 17.82 ± 3.2 | 0.356503 ± 0.063 |
| Ace 50 | 13.96 ± 1.8 | 0.69823 ± 0.088 |
| Ace 100 | 27.19 ± 2.5 | 2.719207 ± 0.250 |
| Ace 150 | 29.66 ± 2.2 | 4.448748 ± 0.324 |
| CAR 0.625 | 18.08 ± 0.4 | 0.011299 ± 0.0002 |
| Car 1.25 | 17.88 ± 0.5 | 0.022348 ± 0.0006 |
| Car 2.5 | 14.72 ± 0.9 | 0.036812 ± 0.0023 |
| Car 5 | 14.57 ± 2.2 | 0.072875 ±0.011 |
| Car 10 | 13.41 ± 1.7 | 0.13409 ± 0.017 |
| Flu 12.5 | 13.69 ± 1.1 | 0.171175 ± 0.013 |
| Flu 25 | 13.01 ± 1.4 | 0.325205 ± 0.034 |
| Flu 50 | 15.78 ± 3.8 | 0.788872 ± 0.188 |
| Flu 100 | 14.77 ± 1.1 | 1.476779 ± 0.107 |
| Flu 200 | 16.03 ± 2.3 | 3.2065 ± 0.461 |


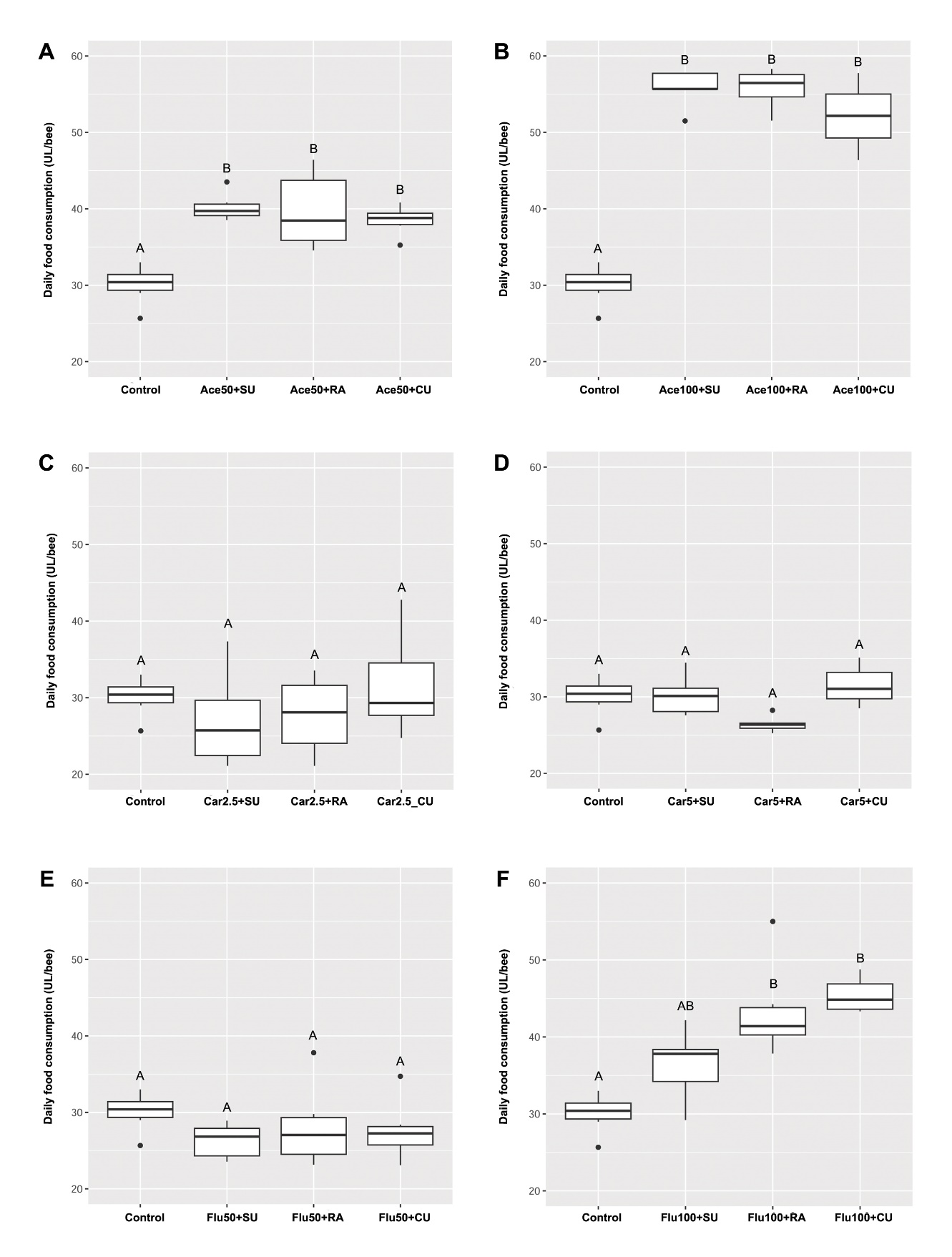


Figure S4- Effect of acetamiprid (A, B), carbaryl (C, D), and flupyradifurone (E, F) and the presence of CU100 and RA100 on daily food consumption of caged honeybees. The control group only received sugar syrup 50%.
